# Research on Schistosomiasis in the Era of the COVID-19 Pandemic: A Bibliometric Analysis

**DOI:** 10.3390/ijerph19138051

**Published:** 2022-06-30

**Authors:** Raquel Sánchez-Marqués, Santiago Mas-Coma, Joaquín Salas-Coronas, Jerôme Boissier, María Dolores Bargues

**Affiliations:** 1 Departmento de Parasitología, Facultad de Farmacia, Universidad de Valencia, Av. Vicent Andres Estellés s/n, Burjassot, 46100 Valencia, Spain; s.mas.coma@uv.es (S.M.-C.); m.d.bargues@uv.es (M.D.B.); 2 CIBER de Enfermedades Infecciosas, Instituto de Salud Carlos IIII, C/Monforte de Lemos 3-5, Pabellón 11. Planta 0, 28029 Madrid, Spain; 3 Tropical Medicine Unit, Hospital del Poniente, Ctra. de Almerimar 31, El Ejido, 04700 Almería, Spain; joaquin.salas.sspa@juntadeandalucia.es; 4 IHPE, University Montpellier, CNRS, Ifremer, University Perpignan Via Domitia, F-66000 Perpignan, France; boissier@univ-perp.fr

**Keywords:** schistosomiasis, COVID-19, bibliometric analysis, socio-economic indicators, correlation analysis, PRISMA

## Abstract

The objectives of this work are to check whether the COVID-19 pandemic affected the research on schistosomiasis, to provide an insight into the most productive countries and journals and the most cited publications, and to analyse any association between the total publications of countries and a set of socio-economic and demographic factors. Based on PRISMA methodology, we used the Scopus database to search for articles published between 1 January 2020 and 26 March 2022. VOSviewer was used to generate the co-authorship and the co-occurrence networks, and Spearman’s rank correlation was applied to study associations. A total of 1988 articles were included in the study. Although we found that the year-wise distribution of publications suggests no impact on schistosomiasis research, many resources have been devoted to research on COVID-19, and the Global Schistosomiasis Alliance revealed the main activities for eradication of schistosomiasis had been affected. The most productive country was the United States of America. The articles were mainly published in *PLoS Neglected Tropical Diseases*. The most prolific funding institution was the National Natural Science Foundation of China. The total publications per country were significantly correlated with population, GERD, and researchers per million inhabitants, but not with GDP per capita and MPM.

## 1. Introduction

The COVID-19, caused by a newly identified strain of the SARS-CoV-2, was officially declared as a pandemic by 11 March 2020 and put additional pressure on health systems around the world. The pandemic heightened inequalities and set back many diseases due to the health policies adopted by governments to prevent the coronavirus transmission. Many studies in high-income countries have claimed that paying less attention to cancer and several chronic diseases, such as cardiovascular and non-infectious respiratory diseases, might lead to avoidable deaths or late diagnostic of lethal diseases [1,2]. This could be especially dramatic for the neglected tropical diseases (NTDs), a diverse group of about 20 diseases endemic in tropical and sub-tropical regions of the globe, affecting more than one billion people, mainly associated with poor hygienic conditions and sanitation facilities and the absence of safe drinking water.

Among the parasitic NTDs, schistosomiasis (also known as snail fever or bilharziasis) is one of the most widespread and the most common parasite transmitted through contact of skin with freshwater contaminated by *Schistosoma* larvae. There are two main forms of schistosomiasis that affect human caused by six main species of blood flukes: intestinal (*S. mansoni*, *S. japonicum*, *S. mekongi*, *S. guineensis*, *S. intercalatum*) or urogenital (*S. haematobium*). The World Health Organization estimates that overall schistosomal infections affect about 240 million people worldwide, of which over 85% live in the sub-Saharan Africa region and nearly 200,000 die every year [3]. Though schistosomiasis is endemic in 52 low-income countries in Africa, Asia, South America, the Middle East, and several Caribbean islands, high-income countries have suffered an increase in imported cases mainly due to migration and international travels and commerce [4,5,6]. For instance, Boissier et al. reported an outbreak of urogenital schistosomiasis in Europe, in Corsica Island (France), produced by parasites imported from Senegal, and alerted the potential risk of schistosomiasis outbreaks in other European areas [7]. European schistosomes had previously been identified as hybrids between the livestock- and the human-infective species *Schistosoma bovis* and *Schistosoma haematobium*, respectively [8,9]. Recently, after an in-depth clinical and epidemiological study of several cases, evidence of autochthonous transmission of urogenital schistosomiasis in Almería (Spain) was demonstrated [10], and new cases of acute schistosomiasis have been reported in people from Antwerp, Belgium after staying in South Africa [11].

The high number of people at risk of getting infected with schistosomiasis makes the need for attention evident. As the best strategy to control and eliminate human schistosomiasis, the World Health Assembly urged State Members to ensure access to regular administration of preventive chemotherapy for the treatment of clinical cases and groups at high risk of morbidity (especially, women and school-aged children) [3]. However, the World Health Organization advised that NTD surveys, active case detection activities, and mass drug administration campaigns should be postponed due to the COVID-19 pandemic, whereas prompt diagnosis should continue if possible. Given this scenario, several studies have pointed up the serious threat of syndemic malaria, NTDs, and COVID-19 in low and middle-income countries [12,13]. In fact, there are warnings that the great advances in the fight against schistosomiasis are at risk of being reversed in many countries due to the measures adopted in the face of the COVID-19 pandemic [14]. It was also evidenced that the interruption of mass drug administration campaigns due to the COVID-19 pandemic would lead to an increase in *S. mansoni* and *S. haematobium* infection [15]. Similarly, the recent outbreak of schistosomiasis reported in northeast Nigeria suggests that one of the possible reasons was the interruption of mass drug administration programs [16]. On the contrary, the number of imported cases of schistosomiasis in non-endemic countries have most likely been affected by the reduced international travel capacity during the COVID-19 pandemic as well as other international travel-related diseases [17,18].

The Global Schistosomiasis Alliance launched a brief questionnaire to some of its partners to know how the COVID-19 pandemic impact on their research activities, revealing that the pandemic and associated restrictions affected clinical studies, field surveillance, and planned basic and preclinical lab work [19]. The purpose of this work is to analyse the scientific literature on schistosomiasis during the COVID-19 pandemic through a comprehensive bibliometric analysis, which may allow us to quantify, measure and visualize the development, potential trends and impact of research on schistosomiasis. This will also shed light on whether the pandemic has affected not only the activities for the prevention, diagnosis and treatment, but also the global research on schistosomiasis.

## 2. Materials and Methods

**Figure 1:**
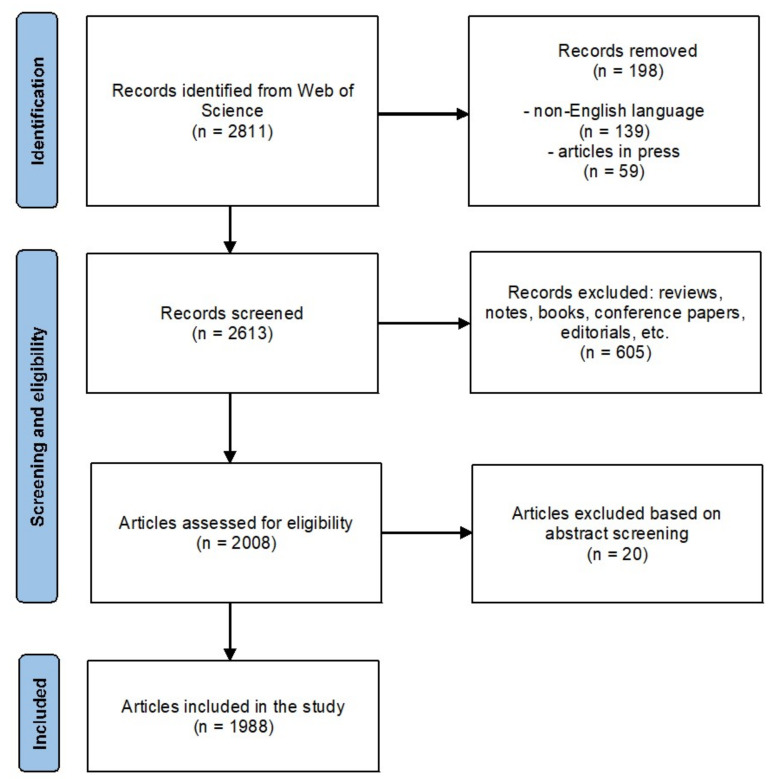
PRISMA flow diagram.

This study was carried out based on the PRISMA statement [20] by cross-searching a comprehensive set of terms (schistosoma, schistosomiasis, snail fever, bilharzia, bilharziasis) in the title, abstract or keywords of an article using the Scopus electronic database for the period from 1 January 2020 to 26 March 2022. Articles were excluded if they met one or more of the following criteria: (i) written in a language other than English, because the impact of publications written in non-English language is usually very low; (ii) reported in conference papers, books, book chapters, editorial material, reviews, conference reviews, short surveys, notes, or errata; and (iii) articles in press. The main reason for excluding these types of publications was that some of them are not usually peer-reviewed, whereas others do not contribute new research (laboratory investigations, clinical studies, etc.). In addition, the abstracts of all identified papers were checked to verify that they were really related to schistosomiasis. The search process was conducted on 27 March 2022. The PRISMA flowchart of the research protocol is shown in Figure 1.

### 2.1. Data Retrieval and Collection

The search strategy identified 2811 records from the Scopus database as it offers an extensive coverage of medical literature [21]. After removing 198 records, 2613 were included for screening. We excluded 605 records based on the document types not considered in this work, leaving 2008 articles to be checked for eligibility. After abstract reading, 20 records were eliminated because they did not meet the inclusion criteria. Finally, a total of 1988 documents were put in the study.

For each paper, meaningful data were collated: Authors, year of publication, title, journal, authors’ affiliation, country, title, keywords, funding/sponsor agency, subject area, and citation count. In addition, we recorded for each paper the number of authors and the country of the corresponding author. These data were then organized in the form of standardized tables to facilitate the analysis of outputs. The impact factor (IF) of journals was gathered from the 2020 Journal Citation Reports, which was the most recent one published by Clarivate Analytics at the moment of preparing this work.

### 2.2. Data Analysis

Spearman’s rank correlation was applied to check for correlation between some bibliometric indices and also between total publications per country and several socio-economic and demographic indicators: population, gross domestic product (GDP) per capita, gross domestic expenditure on R&D (GERD), multidimensional poverty measure (MPM), and researchers per million inhabitants. All these indicators were taken from the websites of the Organisation for Economic Co-operation and Development, the International Monetary Fund, and the World Bank. GERD provides an indication of the level of financial resources devoted to R&D as a percentage of the GDP [22]. MPM is a measure of poverty that captures deprivations in education (attainment and enrolment) and access to basic infrastructure (electricity, sanitation, and drinking water) in addition to the extreme poverty threshold of $1.90 [23].

The Spearman’s correlation coefficient (ρ_s_) was considered significant if the *p*-value was less than 0.05. In addition, we employed VOSviewer [24] tool to perform the analysis of co-authorship and the analysis of co-occurrence of keywords, and *paintmaps.com* to generate a geographical mapping of publications per country.

## 3. Results

**Figure 2:**
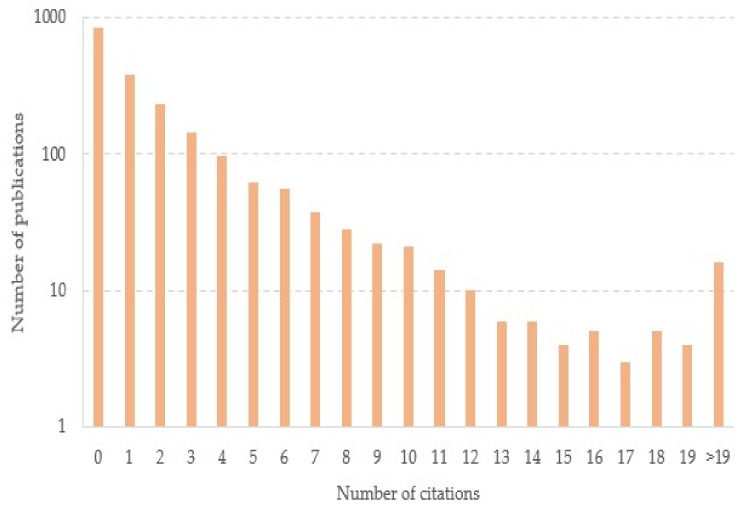
Logarithmic number of publications vs. number of citations.

Out of the 1988 documents included in the study, 1948 were articles and 40 corresponded to letters. These documents were published in 160 different journals and cited 4711 times (as of 27 March 2022). The number of cited documents was 1143 with an h-index of 19. The 159 authors of these publications were from 132 countries on five continents. The overwhelming majority of publications were conducted by multiple authors as only 1.86% of the 1988 documents were sole-authored, leading to a collaboration coefficient of 0.98. The average productivity per year was 662.66 and the average citations per year was 1570.33. The histogram in Figure 2 represents the number of citations against the number of publications in logarithmic scale. The use of a logarithmic scale was to avoid skewing towards large values since the number of publications ranged from 3 (for publications with 17 citations) to 845 (for the case of 0 citations).

### 3.1. Chronological Evolution of Publications

**Figure 3:**
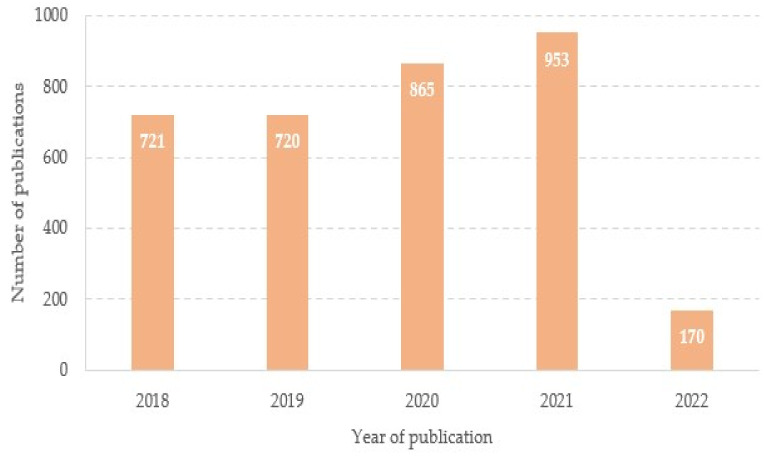
Year-wise distribution of publications.

To examine the trend of publications over time, Figure 3 depicts a distribution bar chart with the number of papers published per year. Here we also included the scores of 2018 and 2019, the two years previous to the pandemic, with the aim of checking whether or not the pandemic caused a decrease in research publications. Surprisingly, visual inspection of this picture revealed that there was a slight increase in the annual number of publications during the acute phase of the COVID-19 pandemic (2020 and 2021), which could suggest that research on schistosomiasis was not dropped out due to the pandemic. However, following the trend of the first three months of 2022, a fairly probable estimate of the number of publications at the end of the year indicates that there could be a certain decrease, probably due to the fact that the resources have been allocated mainly to research on COVID-19 to the detriment of other research lines.

To investigate whether there exists any different trends between systematic reviews and the type of publications included in our study (i.e., those based on experimentation), we also performed a search for reviews published in the period 2018–2022. Thus, the number of reviews from 2018 to 2022 were 141, 155, 184, 219, and 45, respectively. By normal standards, the association between the two variables (research articles versus reviews) should be considered statistically significant (ρ_s_ = 0.9, *p*-value = 0.03739). This suggests that there were no more reviews than experimental articles during the acute phase of the pandemic due to the lockdown and the possible lack of some supplies (e.g., lack of reagents). This could probably be because the experimental articles are based on data and results of experiments carried out during the years prior to the pandemic.

### 3.2. Geographical Distribution of Publications

**Figure 4:**
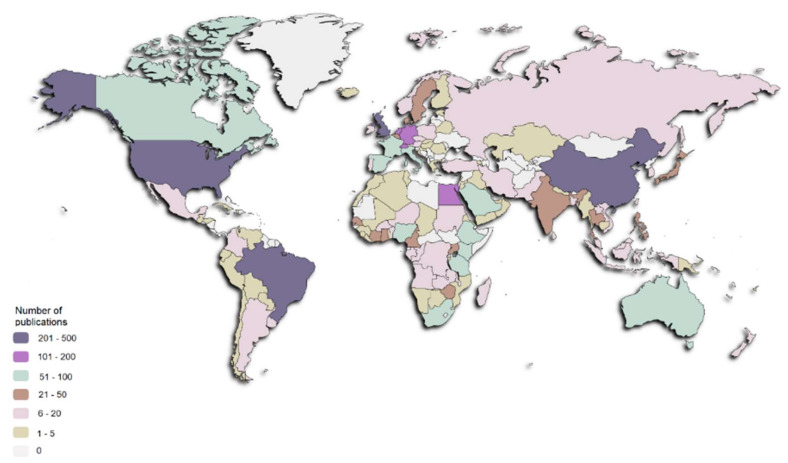
Global mapping of publications on schistosomiasis.

Figure 4 shows a global mapping of the retrieved documents according to the country of all contributing authors. The most active country was the United States of America with 436 publications (21.93%), followed by the United Kingdom (n = 343, 17.25%), China (n = 309, 15.54%), Brazil (n = 245, 12.32%), Egypt and Switzerland (n = 139, 6.99% each), Germany (n = 130, 6.54%), and the Netherlands (n = 106, 5.33%). Authors from 39 countries contributed to the production of only one or two articles.

Focusing on two of the regions most affected by schistosomiasis, we found that the sub-Saharan African countries with the highest number of publications were South Africa (n = 83, 4.18%), Tanzania (n = 80, 4.02%), Ethiopia (n = 73, 7.30%), Nigeria (n = 65, 3.27%), Kenya (n = 62, 3.12%), and Uganda (n = 41, 2.06%), whereas in Southeast Asia, the most productive countries were Thailand (n = 35, 1.76%) and the Philippines (n = 30, 1.51%).

### 3.3. Distribution of Publications by Journals and Research Areas

To analyse the journals that could be considered as the most influential, Table 1 reports the 10 most productive journals together with some bibliometric indices. *PLoS Neglected Tropical Diseases,* with 225 publications (11.32%) and 524 citations, was by far the most used journal in our sample, followed by *Acta Tropica*, with 73 publications (3.67%) and 183 citations. In total, the top 10 journals published 618 documents (31.09%) and received 1601 citations, which accounted for 31.09% of all publications and 33.98% of the total citations. All these journals are ranked in the first quartiles of the 2020 Journal Citation Reports.

The study showed that the number of publications and the number of citations were significantly correlated (ρ_s_ = 0.8628, *p*-value = 0.00131), whereas the number of publications was not significantly correlated with the impact factor (ρ_s_ = 0.4073, *p*-value = 0.24271).

**Figure 5:**
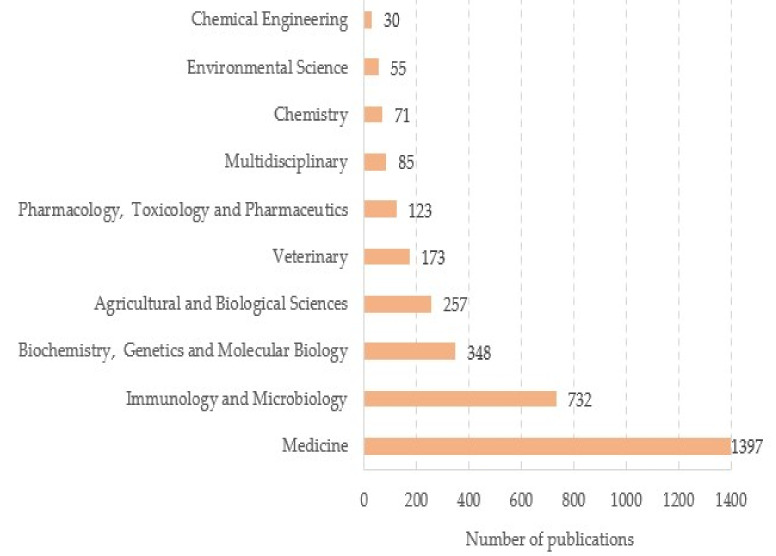
The top 10 research areas.

Regarding the distribution of articles by research areas, we identified a total of 25 different domains. As can be observed in Figure 5, the vast majority of publications belonged to health sciences, such as Medicine (n = 1397, 70.27%), Immunology and Microbiology (n = 732, 36.82%), and Biochemistry, Genetics and Molecular Biology (n = 348, 17.51%). There were less than 30 publications in 15 research fields (e.g., Computer Science, Social Sciences, Nursing, Dentistry, Psychology, Economics, Econometrics and Finance, and Energy). Note that the sum of percentages exceeds 100% because articles could be classified into more than one research area.

### 3.4. Co-Authorship Analysis

**Figure 6:**
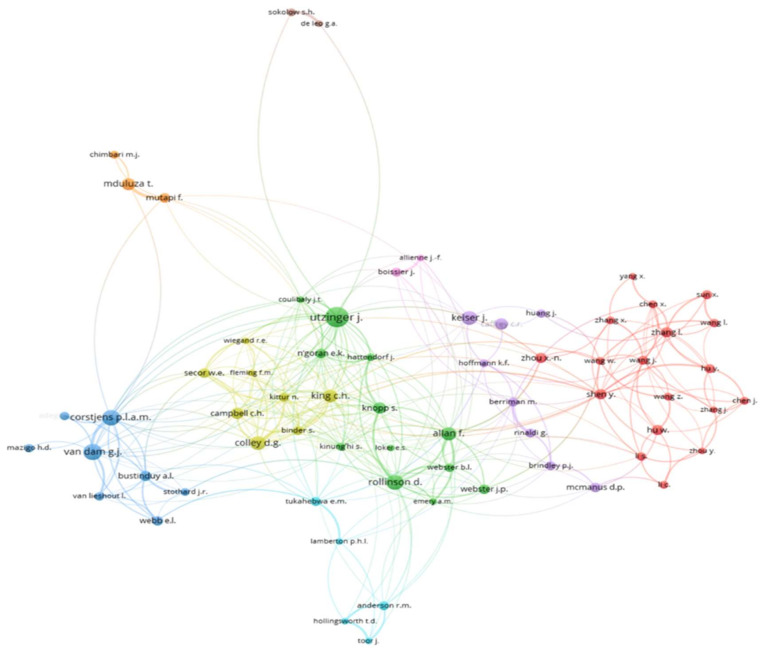
Co-authorship network.

In Figure 6, the co-authorship network highlights the research collaborations between authors. Each node of the network represents an author, and the links between the nodes represent the collaborative relationships between authors. For the sake of a better visualization of the network, only authors with at least 10 documents were selected for this analysis. As can be observed, nine clusters including 83 authors were identified as collaborations in the production of articles. The densest cluster included a total of 29 authors, but with little international collaboration since most of them were from China. There were two clusters formed by only two authors each and one cluster with three authors, showing some kind of inter-institution cooperation. A more exhaustive exploration of the network allowed for finding strong international collaborations between the authors of the different clusters. For instance, we found collaborations between authors from the Swiss Tropical and Public Health Institute with authors from the Natural History Museum of London, the Université de Perpignan via Domitia (France), and the University Félix Houphouët-Boigny (Côte d’Ivoire).

### 3.5. Funding Sources

**Figure 7:**
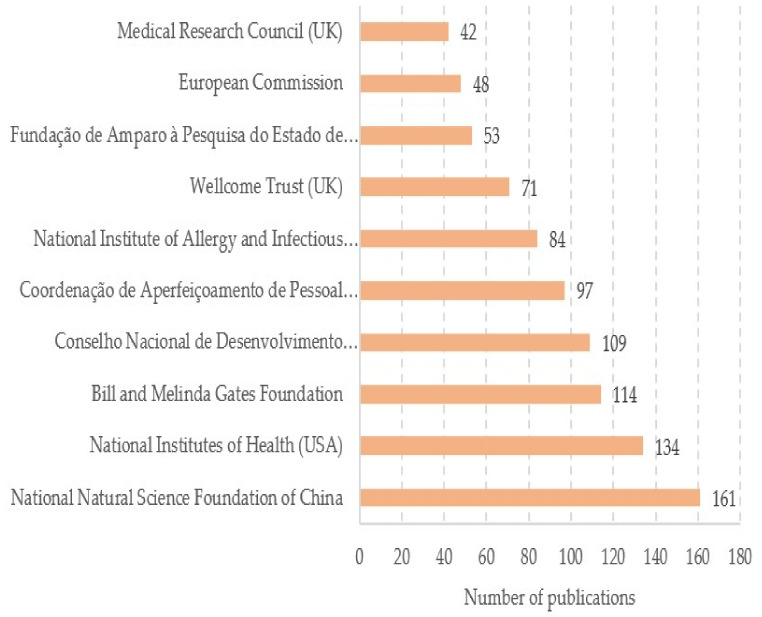
The top 10 funding sources.

A great variety of public and private agencies and institutions were involved in funding the research for the articles included in this study. The most prolific were the National Natural Science Foundation of China (n = 161, 8.10%), the National Institutes of Health USA (n = 134, 6.74%) and the Bill and Melinda Gates Foundation (n = 114, 5.73%). Figure 7 displays the top 10 most common funding sources, which accounted for 45.93% of all retrieved documents.

### 3.6. Co-Occurrence Analysis of Keywords

A co-occurrence network allows for identifying how often a set of keywords appeared together in the publications. In this kind of network, keywords are represented by nodes and their relationships are represented by links; the bigger is the size of a node, the more often the occurrence of a keyword. In addition, the shorter is the distance between two nodes, the stronger their relation.

**Figure 8:**
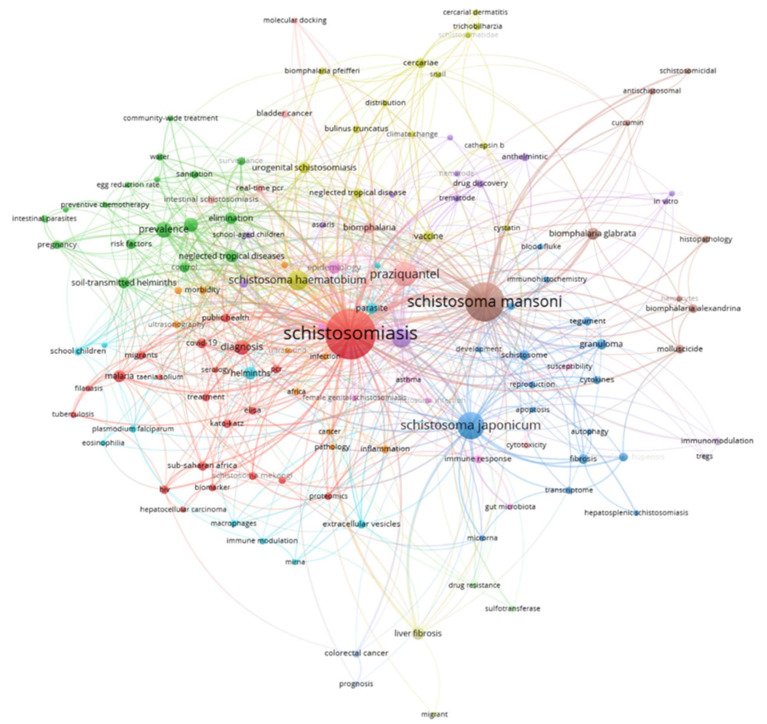
Co-occurrence network of keywords.

Figure 8 shows the co-occurrence network of the retrieved documents in this study. We selected keywords that occurred at least five times to build the co-occurrence network. A total of 14 clusters and 135 items were identified. As expected, “schistosomiasis” was the most representative keyword (360 occurrences), followed by “schistosoma mansoni” (218 occurrences), “schistosoma japonicum” (108 occurrences), “praziquantel” (85 occurrences) and “schistosoma haematobium” (57 occurrences). We also observed the occurrence of other keywords, such as “real-time pcr”, “ultrasonography”, “immune modulation”, “transcriptome” or “biomarkers”, that reveal the current research trends to diagnose the disease.

### 3.7. Most Cited Publications

Table 2 reports the most cited articles ranked by the total citations (TC) and by the field-weighted citation impact (FWCI). The FWCI proposed in Scopus denotes the ratio of the total citations actually received by an article to the average number of citations received by all similar documents over a three-year window; a value equal to 1.00 indicates that the article performs just as expected for the average, whereas a value greater than 1.00 means that the article is more cited than expected according to the average. In addition, this table also shows whether or not an article involves international collaboration (Col) and the number of citations per year (C-Year), which was calculated as: Total citations / (Year of the study—Year of publication + 1).

The top 10 most cited articles by total citations received 590 citations, accounting for 12.52% of the total number of citations (4711). However, it is worth noting that the work ranked as the first accumulated 335 citations, which represents 7.11% of the total. By considering only the remaining top 9 articles, the average number of citations was 28.33 per publication.

The two publications with the highest total citations also received the highest FWCI. However, some important differences were found in these two indices; for instance, the article ranked the third by total citations was the tenth when using the FWCI metric.

Six out of the top 10 most cited articles were performed by authors from different countries. Two articles showed the highest level of international collaboration with authors from nine countries. For instance, the authors of the paper entitled “Circulating anodic antigen (CAA): A highly sensitive diagnostic biomarker to detect active schistosoma infections—improvement and use during SCORE” [34] were from the Netherlands, Switzerland, the United Kingdom, France, Rwanda, Tanzania, Kenya, the United States of America, and St. Lucia.

## 4. Correlation between Total Publications and Country Indicators

According to the Spearman’s correlation coefficient, we found that the total number of publications was significantly correlated with population (ρ_s_ = 0.61196, *p*-value = 0), GERD (ρ_s_ = 0.4629, *p*-value = 0) and researchers per million inhabitants (ρ_s_ = 0.26755, *p*-value = 0.00952). Conversely, the association between total publications and GDP per capita (ρ_s_ = 0.09659, *p*-value = 0.27059) and MPM (ρ_s_ = 0.08238, *p*-value = 0.44018) could not be considered statistically significant. As expected, these results show that research depends mainly on the resources that a country allocates to carry it out, but not so much on the level of wealth of its population. However, more interestingly, this analysis has made it possible to verify that research on schistosomiasis has maintained the same correlations during the COVID-19 pandemic.

## 5. Discussion

Bibliometric analysis constitutes an important tool for exploring the situation on a particular field and offers meaningful information for researchers to evaluate the trends and impact of the research. This work addressed the research on schistosomiasis, which is one of the most widespread neglected tropical diseases, during the COVID-19 pandemic. To the best of our knowledge, this is the first bibliometric analysis with a focus on schistosomiasis research during the pandemic. The bibliometric analysis found that the number of publications increased during the first two years of the pandemic (2020 and 2021), which could be explained because the social lockdown allowed authors more time to write scientific papers in expectation. However, the data for the first months of 2022 suggest a slight slowdown; this change in trend could be due to the fact that many resources have been devoted to research on COVID-19, so that the 2020 and 2021 publications actually refer to research carried out in the pre-pandemic years.

The country with most studies was the United States of America, followed by the United Kingdom, China, and Brazil. Regarding the productivity of regions where schistosomiasis is endemic, the bibliometric analysis revealed that several Sub-Saharan African countries, such as South Africa, Tanzania, Ethiopia, Nigeria, Kenya, and Uganda, contributed significantly to research with a reasonable number of publications. However, when focusing on the Southeast Asia countries, only Thailand and the Philippines had a similar number of articles as those in Africa. Interestingly, most Sub-Saharan African countries have a lower GDP per capita and number of researchers than the Southeast Asian countries, but this does not seem to have been a hinderance for conducting research.

*PLoS Neglected Tropical Diseases*, *Acta Tropica*, and *Parasites and Vectors* were identified as the journals with the largest number of articles on schistosomiasis research in the period analysed. All the most used journals are located in relevant positions in the JCR, although no significant correlation was found between the number of publications and the impact factor. Not surprisingly, the journals with the highest number of articles were in the Tropical Medicine, Parasitology and Infectious Diseases categories of the Journal Citation Reports.

The co-authorship network showed international collaborations and in fact, six out of the top 10 most cited articles were performed by authors from different countries. It is also worth noting that we found collaborations between institutions from countries with high research productivity (e.g., the United Kingdom or Switzerland) and countries with less productivity and high disease prevalence (e.g., Rwanda or Tanzania).

Visualization of the co-occurrence network of keywords highlighted the currently most studied species of blood flukes: *S. mansoni*, *S. japonicum*, and *S. haematobium*. The keyword “praziquantel”, which is a tetrahydroisoquinoline and the drug of choice for the treatment and control of schistosomiasis in many endemic countries because it is effective and its cost is low [35,36], also appeared a high number of times in the documents of our sample. A series of terms associated with diagnosis and immunotherapy development, such as “biomarkers”, “real-time pcr”, “ultrasonography”, and “immune modulation”, were among the most used keywords.

The most cited article was “Global burden of cancer attributable to infections in 2018: a worldwide incidence analysis”, with 335 citations and 111.66 citations per year, which was co-authored by de Martel et al. from France. The field-weighted citation impact of the article was 53.09, demonstrating a significant advantage over the rest of highly cited publications.

Several limitations to this study can be mentioned. First, our analysis was carried out shortly after the end of the acute phase of the pandemic. To have a broader view of the possible impact of the pandemic on schistosomiasis research, this study should probably be repeated when a longer period of time has elapsed from the end of the critical phase of the pandemic in order to have a larger sample of articles. Second, the bibliometric analysis was based on using only the Scopus database and, therefore, some relevant information sources from other bibliographical databases might be omitted from our study.

Another limitation was the exclusion of publications that were not written in the English language. Although there were not many articles written in other languages, this limitation might not reflect the true situation of research on schistosomiasis, especially with regard to the analysis of the geographical distribution of publications. We also excluded meta-analysis because these types of publications were considered not to provide new investigations.

Our study could be further complemented by analysing how the COVID-19 pandemic affected schistosomiasis prevention, diagnosis, and mass drug administration. To this end, a cross-sectional study based on a well-designed structured questionnaire addressed to organizations and institutions that fight against this parasitic disease could provide a more complete perspective and a better understanding of the current situation of control programmes and research on schistosomiasis.

## 6. Conclusions

The aim of this study was to examine the research on schistosomiasis during the COVID-19 pandemic in a holistic manner to discover which are the most active countries, the most widely-used journals and research areas, the main funding sources, and the hot keywords. In addition, we also investigated whether there exists any association between the total number of publications and some socio-economic and demographic factors of the countries.

A total of 1988 documents were included in our analysis. These were published in 160 different journals and cited 4711 times. The number of cited documents was 1143 with an h-index of 19. The 159 authors of these publications were from 132 countries on five continents. The total publications per country were significantly correlated with population, GERD, and researchers per million inhabitants, but not with GDP per capita and MPM.

As pointed out by Hillyer, the main difference between COVID-19 and parasitic diseases is that many of the countries most affected by the current global pandemic have vast economic resources [37]. Therefore, it is essential that the scientific and public health communities fight COVID-19, but not at the cost of neglecting the research on acute and chronic parasitic diseases and their control and prevention programs. In fact, resumption of preventive chemotherapy for schistosomiasis should be carried out immediately because it has also been shown that the administration of praziquantel could reduce active cases of COVID-19 and improve the recovery rate [38].

## Figures and Tables

**Table 1:** Top 10 most prolific journals.

Journal	Articles	Total Citations	Citations per Article	IF
*PLoS Negl. Trop. Dis.*	225	524	2.33	4.411
*Acta Trop.*	73	183	2.51	3.112
*Parasites Vectors*	58	158	2.72	3.876
*Am. J. Trop. Med. Hyg.*	55	244	4.44	2.345
*Front. Immunol.*	42	144	3.43	7.561
*Infect. Dis. Pover.*	36	88	2.44	4.388
*Parasitol. Res.*	36	72	2.00	2.289
*PLoS ONE*	35	62	1.77	3.240
*Pathogens*	30	38	1.27	3.492
*Sci. Rep.*	28	88	3.14	4.380

**Table 2:** The top 10 most cited documents (ranked by total citations and by FWCI).

Rank TC	Rank FWCI	Article	Col ^1^	TC	C-Year	FWCI
1	1	Global burden of cancer attributable to infections in 2018: a worldwide incidence analysis [25]	N	335	111.66	53.09
2	2	Regarding new numerical solution of fractional Schistosomiasis disease arising in biological phenomena [26]	Y(4)	45	15.00	12.52
3	10	A controlled human Schistosoma mansoni infection model to advance novel drugs, vaccines, and diagnostics [27]	N	34	11.33	1.45
4	8	A single-cell RNA-seq atlas of Schistosoma mansoni identifies a key regulator of blood feeding [28]	N	30	10.00	3.08
5	3	Predicted impact of COVID-19 on neglected tropical disease programs and the opportunity for innovation [29]	Y(4)	28	14.00	12.20
6	5	Schistosomal extracellular vesicle-enclosed miRNAs modulate host T helper cell differentiation [30]	N	27	9.00	4.00
7	6	Cancer in Africa 2018: The role of infections [31]	Y(3)	26	8.66	3.74
8	7	Impact of different mass drug administration strategies for gaining and sustaining control of Schistosoma mansoni and Schistosoma haematobium infection in Africa [32]	Y(9)	23	7.66	3.33
9	4	Prevalence and distribution of schistosomiasis in human, livestock, and snail populations in northern Senegal: A one health epidemiological study of a multi-host system [33]	Y(4)	21	7.00	4.88
10	9	Circulating anodic antigen (CAA): A highly sensitive diagnostic biomarker to detect active schistosoma infections—improvement and use during SCORE [34]	Y(9)	21	7.00	3.04

^1^ Y(n): international collaboration (number of countries); N: no international collaboration.

## Data Availability

The data that support the findings of this study are available from the corresponding author upon reasonable request.
